# Methylation of RASSF1A gene promoter and the correlation with DNMT1 expression that may contribute to esophageal squamous cell carcinoma

**DOI:** 10.1186/s12957-015-0557-y

**Published:** 2015-04-08

**Authors:** Zhenzong Du, Kui Ma, Xiaolin Sun, Angui Li, Haiyong Wang, Lifei Zhang, Feng Lin, Xiaoyan Feng, Jianfei Song

**Affiliations:** Department of Cardiothoracic Surgery, Affiliated Hospital of Guilin Medical University, Guilin, 541001 China

**Keywords:** Esophageal squamous cell carcinoma, DNA methylation, DNA methyltransferase 1, RASSF1A

## Abstract

**Background:**

Esophageal squamous cell carcinoma is one of the most common malignancies in the world. Studies have confirmed that there are many genes abnormally hypermethylated in esophageal squamous cell carcinoma. The objective is to detect methylation of the RASSF1A gene promoter and the expression of the DNA methyltransferase 1 (DNMT1) protein in esophageal cancer tissue and discuss their relationship with esophageal squamous cell carcinoma.

**Methods:**

The CpG island methylation status of RASSF1A genes were analyzed in 100 cases of tumor specimens as well as their adjacent tissues which was used for methylation-specific polymerase chain reaction (MSP). The expression of DNMT1 protein was determined by immunohistochemistry. Difference between measurement data and categorical data was compared through analysis of *t* test and chi-square test. All the statistics were taken with a bilateral test. The difference was statistically significant (*P* < 0.05).

**Results:**

The promoter methylation of the RASSF1A gene promoter has been detected in 45 out of 100 (45%) esophageal squamous carcinoma cases, while methylation of RASSF1A gene has been detected in 2 out of 100 adjacent normal tissues (2%). The RASSF1A gene promoter was highly methylated in cancer tissues, and there were significant differences between normal esophagus tissues and esophageal squamous carcinoma (*P* < 0.05). The expression of DNMT1 protein has been detected in 61 out of 100 (61%) esophageal squamous carcinoma cases, including 41 cases in the above 45 methylated samples of RASSF1A gene promoter, and none in adjacent tissues. DNMT1 proteins are highly expressed in cancer tissues, and there were significant differences (*P* < 0.05). In positive cases for methylation of RASSF1A, the DNMT1 protein had been detected in 41 out of 45 (91%), while in non-methylated cancer cases, 20 out of 55(36.3%), and the difference is significant (*P* < 0.05).

**Conclusions:**

Esophageal squamous carcinoma tumorigenesis may be related with hypermethylation of DNMT1 and RASSF1A promoter CpG island due to their high expression and also their hypermethylation.

## Background

Esophageal squamous cell carcinoma is one of the most common malignancies in the world. As a whole, China is considered as the highest incidence country in the world with esophageal squamous cell carcinoma rates being the fourth to sixth of the total malignancies and the second to third of those in the digestive system in China, and it has seriously affected our people’s safety and health [1].The risk factors of esophageal squamous cell carcinoma include chronic nitrosamines stimulation, Western diet(low-fiber, high-fat diet), family history, inflammation and trauma, obesity, and inflammatory esophageal diseases. Studies have confirmed that there are many genes abnormally hypermethylated in esophageal squamous cell carcinoma [2,3]. In order to find new a diagnostic molecular marker in esophageal squamous cell carcinoma, this study focuses on the expression of the key enzyme in the process of DNA methylation - DNA methyltransferase 1 (DNMT1) - in esophageal squamous cell carcinoma tissues and the relationship with the promoter methylation status of CpG island in RASSF1A.

## Methods

### The clinicopathologic data

With the informed consent of all patients and approval of the ethics committee, tumor tissues were obtained during surgery, and the diagnosis of esophageal squamous cell carcinomas was confirmed by HE staining from 100 patients at the affiliated Hospital of Guilin Medical College from 2002 to 2012 (The affiliated hospital of Guilin medical University ethics committee gives approval to this study). Clinical-pathological features are shown in Table 1. No patients had received preoperative chemotherapy, radiation therapy, or other biological therapy. The clinical records and pathological data were of good integrity (Table 1).

**Table 1:** **Clinical-pathologic profiles of the esophageal squamous cell carcinoma cases in this study**

**Clinical-pathologic features**		**Number of cases**
Gender	Male	72
	Female	28
Age (year)	Median	63.4
	Range	42 to 74
Tumor site	Upper	12
	Midpiece	74
	Lower	14
Histological types	Squamous cell carcinoma	100
Differentiation	Moderate	82
	Poor	18
Follow-up 27 (3 years)	Survival	86
	Deceased	14
TNM stage	0	2
	I	11
	IIa	37
	IIb	39
	III	9
	IV	2

TNM, tumor node metastasis.

### Main reagents

The main reagents were the DNA rapid extraction kit from Aidlab Biotechnologies Co., Ltd. (Haidan District, Beijing), the EZ Bisulfite DNA Clean-up Kit from Aidlab Biotechnologies Co., Ltd. (Haidan District, Beijing), the PCR Amplification Kit from Aidlab Biotechnologies Co., Ltd. (Haidan District, Beijing), and the DNMT1 Monoclonal Antibody from (Sigma-Aldrich, Shanghai, China).

### MSP

Genomic DNA (1 μg) was harvested with a DNA rapid extraction kit for bisulfite modification with the CpG genome kit according to the manufacturer’s instructions; 5 μL of bisulfite-modified DNA was used per 25 μL methylation-specific PCR (MSP) reaction. For PCR, methylated (M) and unmethylated (U) primer pairs were initially denatured at 94°C for 3 min followed by 35 cycles with a 1-min denaturation step, 30 s of annealing at 60°C( demethylation 58°C), and 3 min of extension at 72°C. Final extension after 35 cycles was at 72°C for 10 min, and the product was stored at 4°C. PCR products were analyzed by agarose gel electrophoresis and ethidium bromide staining, and the objective gene stripes were used as the positive expression. RASSF1A-U used the methylation-specific primers 5′-AACATAACCCAATTAAACCCATACTTC-3′(sense)and 5′-GGGGTTTGTTTTGTGGTTTTGTTT-3′(antisense) (product length 105 bp) and RASSF1A-M using the unmethylation-specific primers 5′-GGGTTCGTTTTGTGGTTTCGTTC-3′(sense)and 5′-TAACCCGATTAAACCCGTACTTCG-3′(antisense) (product length 105 bp), plus 100 ng of bisulfite-modified DNA.

### Immunohistochemistry analysis

All of the tissues were fixed in 10% neutral formalin, embedded in paraffin, and serial sectioned into 4 μm. The antibody dilution ratio is 1:200. Using the known positive section as the positive criteria, PBS displaces the primary antibody as the negative criteria. The immunostaining was performed with the antibody using DAKO Envision system/3,3-diaminoben-aidine (DAB) staining. The result was scored by conjunction with both staining intensity and the percentage of positive staining cells. To identify the IHC, all the made tissue sections were stained with H&E and double blind was used in the experiment. Each section was reviewed by two pathologists independently, and the results were reconfirmed once inconsistent. Semi-quantitative integral analyses were used to estimate the results, and each sample was given an intensity score (0 to 3) and a percentage of cell positive score (0 = less than 5%, 1 = 6% to 25%, 2 = 26% to 50%, 3 = 51% to 75%, 4 = more than 75%).An overall immunohistochemistry score was calculated by multiplying the intensity and percentage of cell positive scores. Canary yellow colors were handled as 1 point, claybank as 2 points, and brown as 3 points. Scores of 0 were recorded as negative, 1 to 4 as weakly positive (+), 6 to 8 as positive (++), and 9 to 12 as strongly positive (+++). Furthermore, we counted the negative and weakly positive as ‘negative’ and the positive and strongly positive as ‘positive’.

## Results

### The comparison of promoter methylation of RASSF1A gene between esophageal carcinoma tissues and the para-cancerous tissues

**Figure 1:**
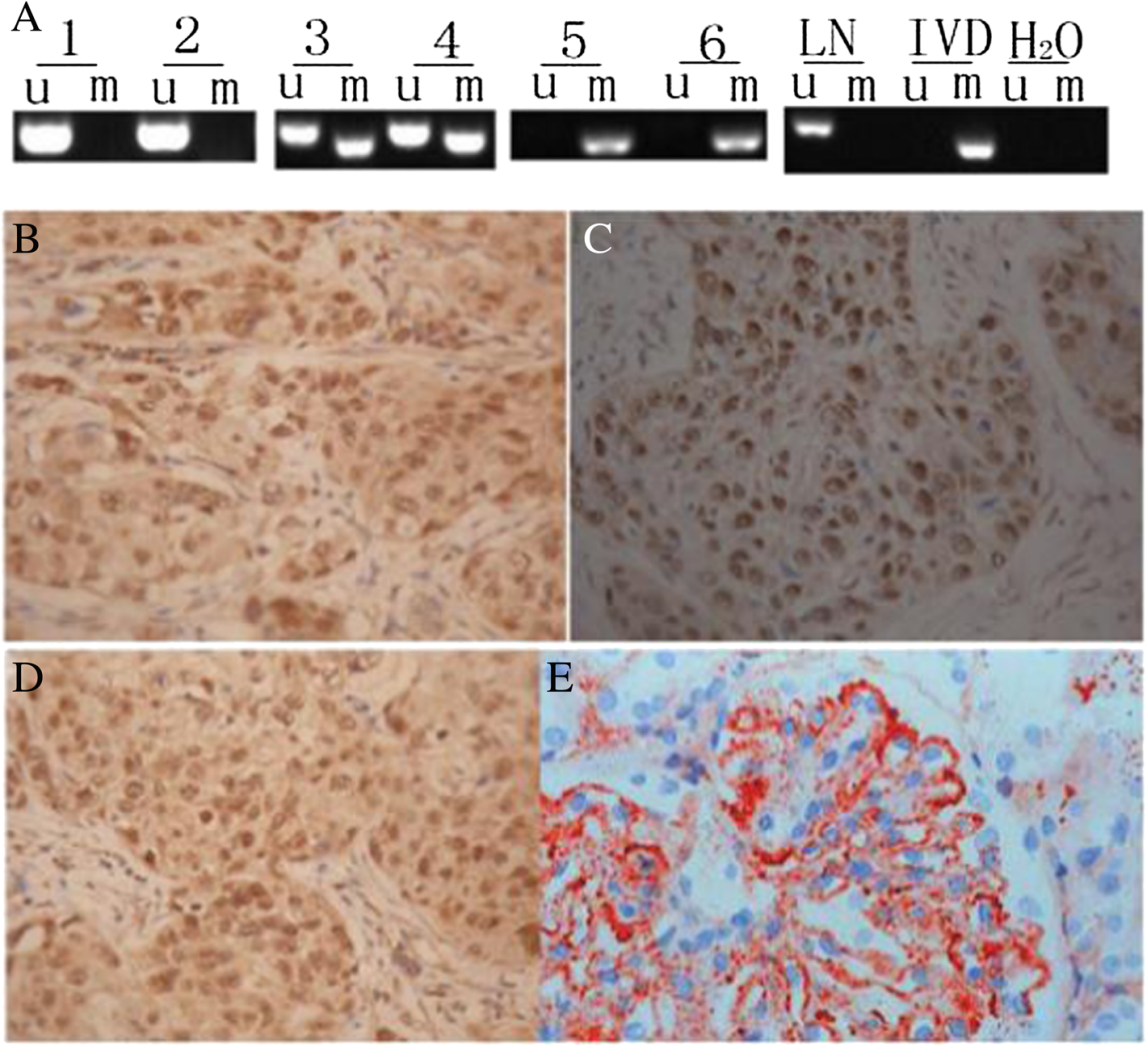
**Representative MSP results in tumor tissues and the expression of DNMT1 protein in esophageal squamous carcinoma tissue. (A)** Representative MSP results in tumor tissues: 1, 2, 3, 4, 5, and 6 for tumor tissues; 1 and 2 represented unmethylation which was the demethylation positive control; 4, 5, and 6 embodied hypermethylation and was the methylation positive control. IVD, *in vitro* DNA methylation; u, unmethylation; m, methylation. **(B-D)** The expression of DNMT1 protein in esophageal squamous carcinoma tissue by immunohistochemistry results (SP× 400). **(B)** Weakly positive; **(C)** positive strongly positive; **(E)** normal esophageal tissue, negative control.

Among the 100 patients, the promoter methylation of RASSF1A gene (overall or partly) was found in 45 patients (45%). And the promoter methylation of RASSF1A gene was also detected in adjacent normal tissues (2%). The RASSF1A gene promoter was highly methylated in cancer tissues, and there were significant differences between normal esophagus tissues and esophageal squamous carcinoma (*P* < 0.05) (Figure 1A).

### The different expression of DNMT1 in normal esophagus tissues and esophageal squamous carcinoma was detected by immunohistochemistry

The express of DNMT1 protein has been detected in 61 out of 100 (61%) esophageal squamous carcinoma cases and none in adjacent tissues. Among the 45 cases whose RASSF1A gene promoter CpG island methylation or partial methylation was detected in the MSP experiments, it was found that DNMT1 was expressed positively in 41 cases. DNMT1 proteins are highly expressed in cancer tissues, and there were significant differences (*P* < 0.05) (Figure 1B,C,D).

### The relationship between the promoter methylation of RASSF1A gene and the expression of DNMT1 protein in esophageal squamous carcinoma

In the CpG-island-methylated esophageal squamous carcinoma cases, the DNMT1 protein has been detected in 41 out of 45 (91%), while in non-methylated cancer cases, 20 out of 55(36.3%), and the difference is significant (*P* < 0.05) (Table 2).

**Table 2:** **Relationship between promoter methylation of RASSF1A gene and expression of DNMT1 protein in esophageal squamous carcinoma**

**Parameters**	**DNMT1**		**Total**	**Positive incidence (%)**
	**Positive**	**Negative**		
RASSF1A	*			
Methylation	41 (total contained in *)	4	45	91
Demethylation	20	35	55	36.3
Total	61	39	100	61

*Represents the 45 cases of promoter methylation of RASSF1A gene.

## Discussion

As a new tumor suppressor gene, RASSF1A gene was cloned from the three short arms of a chromosome (3 p21. 3) in 2000, and it is located at 3 p21. 3 on a 120-kb-long minimum homozygous loss zone. Because of the differences between alternative splicing and promoter, it formed three main transcripts, called A, B, and C. The cDNA’s length of RASSF1A gene was 1,837 bp, including the open reading frame encoding formed by 340 amino acids which consisted of six exons together in sequence. The protein weight by RASSF1A gene transcription was 38.8 kD, whose N-terminal binding area with diacylglycerol and phorbol esters was highly homologous. The N-terminal binding area is also regarded as the conservative area of the protein kinase C, and the C-terminal of the protein originated same as with the Ras-effecting protein Nore1 and rat Maxp1 of the mouse. The RASSF1A gene can regulate cell proliferation and apoptosis by inhibiting the Ras/RASSF1/ERK signal transduction pathways, as well as maintain the stability of microtubules [4]. Previous studies have reported the involvement of RASSF1A promoter methylation in several cancers, including prostate [5], ovarian [6], endometrial [7], gastric [8], lung [9], and breast cancer [10]. And, it has been generally accepted that the hypermethylated status of the promoter CpG islands represents the long-term transcription-silencing state of the genes [11-13].Other researches have also offered the evidence that RASSF1 stimulation of lung cancer cell proliferation depends on IGFBP-5 and PIWIL1 expression levels [14].

In this research, we found that the RASSF1A gene promoter was highly methylated in cancer tissues, and there were significant differences between normal esophagus tissues and esophageal squamous carcinoma. It was conjectured that the promoter CpG island hypermethylation of RASSF1A in human esophageal squamous carcinoma plays a powerful role in the inactivation and transcriptional gene silencing of the RASSF1A gene. But it does not exist in esophageal RASSF1A promoter methylation, and the gene expressed negatively in several patients with the possible mutual adjustment of a bit of a mutation and loss of heterozygosity.

Not only did we focus on the status of RASSF1A methylation, but we also explored its possible mechanism preliminarily. As one member of DNMTs’ family, DNMT1 has the close relationship with gene methylation in the present study, which can maintain the gene’s methylation. DNMT1 is the predominant methyltransferase, functioning primarily to maintain DNA methylation patterns after DNA replication. Research [15] has shown that the expression DNMT1 was high in human esophageal squamous carcinoma, suggesting that DNMT1 could regulate the key gene’s function abnormally in human esophageal squamous carcinoma. DNMT1 expression abnormalities may be involved in the process of esophageal cancer, but the development in the majority of esophageal cancers is related to multiple factors, genes, and multiple-step processes. Its pathogenesis is very complex and not yet clear. Additional studies [16] have demonstrated that DNMT3b is also expressed in healthy tissues, albeit at levels considerably lower than DNMT1, and function primarily in *de novo* methylation. Specific inhibition of DNMT1 or DNMT3b expression promotes growth arrest in cancer cells and attests to the relevance of DNMT expression-activity during malignant transformation [16]. DNMT3b may participate in gene methylation in multiple tumors, which can be used as early biomarkers in the early diagnosis of a tumor [16]. Results of our studies clearly indicate that the DNMT1 protein is highly expressed in cancer tissues, and there were significant differences. And furthermore, we also found that in the positive cases for methylation of RASSF1A, the DNMT1 protein had overexpressed in non-methylated cancer cases with significant difference (41 DNMT1 positive cases in 45 RASSF1A positive cases). With the comprehensive results above, we could presume that DNMT1 high expression in esophageal cancer tissues could lead to high methylation in RASSF1A gene promoter CpG island. This is only a preliminary speculation of this experiment; its reason and mechanism is not clear and further investigation is expected. DNMT1 and RASSF1A gene high expression in cancer patients, perhaps, can be used as a tumor marker for the early diagnosis of tumors [17].

Esophageal carcinoma is the eighth most common cancer in the world and the seventh leading cause of cancer death worldwide [18]. Although surgical treatment to esophageal cancer is the main method currently, early diagnosis and operation is the keystone in reducing the mortality of esophageal cancer. Epigenetics may play an important role in tumor diagnosis and treatment gradually.

## Conclusion

In conclusion, the high expression of DNMT1 in esophageal cancer compared with normal tissue probably reflects the increased methylation of RASSF1A gene promoter CpG island. And, the expression quantity of DNMT1 increased accompanied with the elevating degree of its hypermethylation. DNA methylation is one of the important contents in epigenetics research, and it is closely related to the occurrence and the development of various tumors with tissue specificity. On the basis of prophase studies, future research should peg where certain methylation differences are associated with esophageal cancer, establish the esophageal-cancer-related gene methylation spectrum, and focus on DNMT’s family roles and specific mechanisms. Thus, we could provide epigenetics theoretical basis of the diagnosis and treatment for esophageal squamous carcinoma.

## Consent

Written informed consent was obtained from the patient for publication of this paper and accompanying images. A copy of the written consent is available for review by the Editor-in-Chief of this journal.
